# A survey on the resources and practices in pediatric critical care of resource-rich and resource-limited countries

**DOI:** 10.1186/s40560-015-0106-3

**Published:** 2015-10-09

**Authors:** Sandeep Tripathi, Harsheen Kaur, Rahul Kashyap, Yue Dong, Ognjen Gajic, Srinivas Murthy

**Affiliations:** Division of Pediatric Critical Care Medicine, Mayo Clinic, Rochester, MN USA; Division of Pulmonary and Critical Care Medicine, Mayo Clinic, Rochester, MN USA; University of British Columbia, Vancouver, British Columbia Canada; Department of pediatrics, University of Illinois at Chicago, Peoria, IL USA

**Keywords:** Pediatric critical care, International survey, Low- and middle-income countries

## Abstract

**Background:**

Contemporary critical care research necessitates involvement of multiple centers, preferably from many countries. Adult and pediatric research networks have produced outstanding data; however, their involvement is restricted to a small percentage of the industrialized nations. Implementation of their findings in low- and middle-income countries (LMICs) is fraught with challenges.

**Methods:**

We conducted an online international survey to assess and compare disease burden and resources to participate in multicenter research studies through a listserv of the World Federation of Pediatric Intensive and Critical Care Societies. Respondents were grouped into high-income countries and LMICs on the basis of World Bank classification.

**Results:**

Survey was completed by 73 centers in 34 countries (34 from high-income countries and 39 from LMICs). Compared with high-income countries, the pediatric intensive care units in LMICs were characterized by a lower number of critical care specialists, more difficult access to hemodialysis, and a lower number of elective postoperative patients, but a similar overall disease burden. Training and resources for research were comparable in the two cohorts.

**Conclusions:**

Although differences exist in access to both trained providers and equipment, the survey results were more striking in their similarity. It is essential that centers from LMICs be included in multinational studies, to generate results applicable to all children worldwide.

## Background

The delivery of pediatric critical care (CC) in resource-rich regions has progressed dramatically over recent decades, with markedly improved outcomes for critically ill children around the planet. Many of the advances in the care of critically ill children have been through high-cost interventions, with enhanced accessibility to resources, trained staff, and new technology. However, the vast majority of the world’s child population and pediatric disease burden are in regions where resource limitations are more prevalent [1]. There are scant data about the nature of global pediatric CC, especially in resource-limited regions. Given the infrastructure required to maintain a CC unit, ensuring personnel and equipment availability is a tremendous challenge in resource-limited economies.

A few reports have addressed the adult CC services and utilization in developing countries [2]. However, little is known about the international differences in pediatric CC services. Although individual countries have previously published data on the resources [3, 4], no large international study has compared resources among the different parts of the world. In addition, much of the CC evidence base has come from resource-rich countries [5]; research from low- and middle-income countries (LMICs) represents only a small fraction of CC research performed around the planet [6–8].

Incorporation of evidence from different regions of the world with different health systems is fraught with difficulty. Evidence generated within a sole country or across countries is often implemented in the care of critically ill patients worldwide without further local testing [9]. Research done in resource-rich regions, when reproduced in other countries, has led to contradictory results [10]. Therefore, it is essential that high-quality research be carried out in regions where disease burden is high, to apply locally generated evidence.

The present survey was conducted to identify the pediatric CC resources and to research the infrastructure available in resource-limited countries as part of recruitment efforts for a multicentric research study to evaluate the automated rounding and clinical decision support tool, Checklist for Early Recognition and Treatment of Acute Illness in Pediatrics (CERTAINp) [11]. We describe herein the secondary data analysis of survey results.

## Methods

We adapted a survey for pediatric CC providers from a previously published and validated Web-based survey [12]. The resulting cross-sectional survey consisted of 138 questions containing numeric, binomial, and categorical questions, as well as descriptive questions. The survey was administered to email addresses obtained from the email list of the World Federation of Pediatric Intensive and Critical Care Societies. This list contained more than 6000 persons (physicians, nurses, midlevel providers, and other health care professionals) active in pediatric intensive care from around the world, with representation from every inhabited continent. The survey was conducted in November and December 2014.

Respondents were asked to classify the various diseases and conditions in the intensive care unit (ICU) on a spectrum of *very common* to *very uncommon*, including rank-order lists of causes of death in their hospital. Access to CC resources, such as equipment, skilled staff, and medications, was categorized on a scale from *very difficult* to *very easy*. All answers were based on the perceptions of the respondent individually. Hospital location was classified as *resource-rich* or *resource-limited* on the basis of World Bank classification [13]. High-income (gross national income per capita, >US$12,746) countries (HICs) were classified as *resource-rich*. By comparison, the other areas were classified as upper-middle-income countries (UMICs) (gross national income, US$4126–US$12,745 in 2013), LMICs (gross national income, US$1046–US$4125), and low-income countries (LICs) (gross national income, ≤US$1045) as *resource-limited*.

### Statistical analysis

Raw data from the survey were exported to an electronic file for analysis (JMP; SAS Institute Inc.). Numeric values, such as the number of pediatric beds in a hospital or the number of intensivists, were analyzed as mean (SD) and compared between the two groups with *t* test. Binomial variables such as presence or absence of a research coordinator or of formal CC training were analyzed with *χ*^2^ test as proportions. Categorical variables with more than two choices were dichotomized into two groups for analysis with *χ*^2^ test. Because of the large number of questions, certain related fields were grouped together for analysis. The study was approved by the Mayo Clinic Institutional Review Board as part of the multicentric CERTAINp study. Need for informed consent was waived. There were no patients enrolled in the survey and no patient-specific data was collected.

## Results

The survey was emailed twice—on November 5 and November 27, 2014—to 6172 and 6141 respondents. The emails were read by 1838 and 1742 recipients, with 326 and 245 opening the survey. A total of 73 hospitals (12.9 % response rate) completed the survey, representing 34 countries: 15 HICs, 14 UMICs, 3 LMICs, and 2 LICs (15 resource-rich and 19 resource-limited). All responses were from separate institutions. Pediatric age range in the earlier study and suggested for this survey was birth to 18 years.

### ICU and research capacity

The mean (SD) total numbers of pediatric beds were comparable in the two groups (*P* = .79) (Table 1). The numbers of self-defined CC beds (*P* = .007) and ICU admissions (*P* = .03) were significantly greater in HICs. Among centers in resource-limited regions, 51.2 % had a research coordinator and 66.6 % had a dedicated quality improvement program. No difference was found in the availability of reliable Internet access in the ICU between the two regions. Resource-limited regions also had limited implementation of electronic medical records (*P* = .001). A higher proportion of centers from HICs had participated in prior multicentric trials; however, this difference was not statistically significant (*P* = .13).

**Table 1:** Comparison of the resources between two groups of countries in accordance with survey responses

Resource^a^	Developed country (*n* = 34)	Developing country (*n* = 39)	*P* value
Pediatric beds	169 (88.4)	158 (211)	.79
Critical care beds	17.5 (11.5)	11.2 (6.8)	.007
Admissions/year in PICU	870.4 (548)	600.1 (504)	.03
PICU physicians	10.0 (5.8)	5.5 (4.3)	<.001
EMR	85.29 (29)	48.72 (19)	.001
Dedicated research coordinator	47.06 (16)	51.28 (20)	.72
Dedicated QI program	76.47 (26)	66.67 (26)	.36
Reliable Internet access	97.06 (33)	87.18 (34)	.13
Critical care specialization	94.12 (32)	94.87 (37)	.89
Prior participation in multicentric trials	85.2 (29)	58.9 (23)	.13
ACLS/PALS	91.18 (31)	82.05 (32)	.26
PFCCS	32.35 (11)	35.90 (14)	.75

*ACLS* advanced cardiac life support, *EMR* electronic medical record, *PALS* pediatric advanced life support, *PFCCS* pediatric fundamental critical care support, *PICU* pediatric intensive care unit, *QI* quality improvement

^a^Numerical values are presented as number (SD); categorical values are presented as percentage and number of “Yes” responses

### ICU staffing

Almost twice the number of intensivists (i.e., physicians primarily practicing in the pediatric intensive care unit) work in each unit in the resource-rich countries (mean [SD], 10.0 [5.8] vs 5.5 [4.3]). However, there was comparable staff (doctor or nurse) with CC training (CC specialization, advanced cardiac life support, pediatric advanced life support, or pediatric fundamental CC support; *P* = .89) (Table 1). Access to the different services of CC delivery was not reported as *difficult* or *very difficult* in industrialized countries, and ICU-trained nurses and occupational and physical therapists were significantly less readily available in resource-limited regions (23.08 and 17.95 % reporting difficult or very difficult, respectively) (Table 2). Among all the support services, respiratory therapists were the most difficult to access, even in HICs and LMICs (reported *difficult* or *very difficult*, 20.59 vs 38.46 %; *P* = .09). An anesthesiologist was the only medical provider with a significant difference in difficulty to access between the two groups: No center from resource-rich countries reported difficulty, whereas 12.82 % from resource-limited regions reported either *difficult* or *very difficult* access.

**Table 2:** Difficulty in the availability of trained staff and finances

Trained staff^a^	Developed country, % (no.) (*n* = 34)	Developing country, % (no.) (*n* = 39)	*P* value
Anesthesiologist	0 (0)	12.82 (5)	.03
Surgeon	0 (0)	2.56 (1)	.35
ICU specialist	0 (0)	7.69 (3)	.10
Nurse	0 (0)	2.56 (1)	.35
ICU-trained nurse	0 (0)	23.08 (9)	.002
Pharmacist	2.94 (1)	12.82 (5)	.13
OT/PT	2.94 (1)	17.95 (7)	.04
RT	20.59 (7)	38.46 (15)	.09
Finances	2.94 (1)	35.90 (14)	<.001

*ICU* intensive care unit, *OT* occupational therapist, *PT* physical therapist, *RT* respiratory therapist

^a^Values indicate the percentage of respondents in the group who selected “difficult” or “very difficult” for the survey item

### ICU resources

Although all survey-included ICU equipment was harder to access in resource-limited countries, only central intravenous catheter (*P* = .03), ICU monitoring equipment (*P* = .01), radiology services (*P* = .009), and dialysis equipment (*P* < .001) were statistically significant (Table 3). No difference was found in access to hospital beds, ICU beds, operating rooms, mechanical ventilators, or cardiac defibrillators. Only three centers from the LMIC group reported difficulty in access to crystalloid fluids (normal saline or lactated Ringer solution). Accessing antimicrobials was difficult in 25.6 % of resource-limited regions, compared with 14.7 % of resource-rich regions. Cardiovascular medications were difficult to access in 2.5 % of resource-limited regions vs 0 % in resource-rich regions. Opioids, benzodiazepines, and ketamine were significantly more difficult to access in resource-limited regions (23.08 vs 2.94 %, *P* = .01). For 23.08 % of respondents in resource-limited regions, blood products were difficult to obtain. Finances continue to be a challenge in these regions as well, with 35.9 % of the centers struggling for adequate financial resources compared with 2.9 % in HIC reporting difficulty with finances (data not shown).

**Table 3:** Difficulty in the availability of ICU equipment and medications

Equipment and medication^a^	Developed country (*n* = 34)	Developing country (*n* = 39)	*P* value
Hospital beds	0 (0)	10.26 (4)	.05
ICU beds	2.94 (1)	12.82 (5)	.13
Operating room	0 (0)	5.13 (2)	.10
Central IV catheter	0 (0)	12.82 (5)	.03
ICU monitoring devices	2.94 (1)	23.08 (9)	.01
Mechanical ventilators	0 (0)	7.69 (3)	.10
Cardiac defibrillators	0 (0)	5.13 (2)	.18
Radiology equipment	0 (0)	17.95 (7)	.009
Dialysis equipment	2.94 (1)	38.46 (15)	<.001
Blood bank	0 (0)	5.13 (2)	.18
Antimicrobials	14.71 (5)	25.64 (10)	.25
Pain/sedation medications^b^	2.94 (1)	23.08 (9)	.01
Cardiovascular drugs	0 (0)	2.56 (1)	.35
Crystalloid fluids	0 (0)	7.69 (3)	.09
Blood products/albumin	0 (0)	23.08 (9)	.002

*ICU* intensive care unit, *IV* intravenous

^a^Percentage of respondents in the group who selected “difficult” or “very difficult” for the survey item

^b^These medications include opioids, benzodiazepine, and ketamine

### Admitting diagnosis and causes of death

Malnutrition continues to be prevalent in resource-limited countries, as suggested by 35.9 % of the countries reporting very frequent nutritional diseases in the ICU (Table 4). Tuberculosis (28.1 %), malaria (10.2 %), human immunodeficiency virus (10.2 %), and rheumatic heart disease (7.7 %) were specific to resource-limited regions. The ICUs of resource-rich regions reported a much higher proportion of patients receiving an elective operation. Respiratory disease and congenital heart disease were the two diseases most frequently cited as very common in both resource-rich and resource-limited regions, representing 88.24 and 87.18 % for respiratory disease and 44.12 and 43.59 % for congenital heart disease, respectively. The top 3 causes of death reported by respondents were similar across income groups: infection, multiorgan dysfunction, and cardiac reasons. Infectious causes of death comprised a greater percentage in the resource-limited countries than resource-rich countries (51.28 vs 23.53 %, *P* = .25) (data not shown).

**Table 4:** The most common disease states cited by survey respondents

Disease state^a^	Developed country (*n* = 34)	Developing country (*n* = 39)	*P* value
Toxins	11.76 (4)	12.82 (5)	.89
Newborn illness	41.18 (14)	30.77 (12)	.35
Tuberculosis	0 (0)	28.21 (11)	<.001
Malaria	0 (0)	10.26 (4)	.05
HIV infection	0 (0)	10.26 (4)	.05
Rheumatic heart disease	0 (0)	7.69 (3)	.10
Congenital heart disease	44.12 (15)	43.59 (17)	.96
Nutritional disease	.05 (0)	35.90 (14)	<.001
Cancer	35.29 (12)	17.95 (7)	.09
Respiratory disease	88.24 (30)	87.18 (34)	.89
Elective operation	64.71 (22)	33.33 (13)	.007
Emergency operation	47.06 (16)	30.77 (12)	.15
Renal failure	26.47 (9)	17.95 (7)	.38
Diabetes mellitus	23.53 (8)	7.69 (3)	.06
Status epilepticus	23.53 (8)	12.82 (5)	.23
Sepsis	26.47 (9)	20.51 (8)	.55
Meningitis	11.76 (4)	23.08 (9)	.21

*HIV* human immunodeficiency virus

^a^Percentage of respondents in the group who answered *very common* to the particular disease or syndrome

## Discussion

This study reports both large variation and remarkable similarities in CC services across the 34 countries. Pediatric CC providers all over the world continue to struggle with infectious diseases and congenital heart disease. From a research standpoint, many of the survey participants in resource-limited regions appear to have the capacity to participate in clinical research. The lack of resources and higher incidence of infectious disease-related deaths in developing countries are expected based on higher birth rate and financial constraints. Our finding of deficits in certain areas, such as staffing and medication access, in resource-limited regions should help guide policy in CC delivery in these regions.

In the past, several attempts were made to identify overall similarities and differences in CC services; however, most of the studies were limited to a comparison of centers in developed countries [14] or individual countries [15]. International comparisons of pediatric CC delivery have been hampered by the lack of internationally validated outcome prediction scores, given that the primary scores have been validated in resource-rich regions only [16]. Their application to ICU services in varied settings with varied diseases and clinical contexts leads to challenges in interpretation.

A previously published international survey on adult ICU resources in developing countries showed that in a convenience sample of 13 ICUs from resource-limited regions, similar ICU capacities were found (median, 9 beds occupied and 40 patients treated per month; mean [SD] beds, 11.2 [6.8]; mean [SD] admissions per year, 600 [504]), although no resource-rich region comparison occurred [17]. They also observed that standardized processes of care (e.g., checklists) are frequently lacking in these countries. We did not compare specific outcome data or severity of illness among regions, although prior studies have shown higher mortality rates and younger age groups in adult CC settings in resource-limited regions [7]. As pediatric CC grows increasingly, the capacity in many regions of the world—such as Asia and Africa—to incorporate these data will allow for better evidence guidance.

Given the variability in insurance schemes and health financing in resource-limited regions, CC may be out of reach for most patients who currently are in resource-limited regions [18]. However, cost-effective CC, where more-than-usual resources are devoted to rescuing severely ill patients, has a fundamental place in any health system and should be a fundamental goal of the field of pediatric CC [19]. This is especially true in pediatrics, where the potential for recovery after acute illness is high and the disease burden is substantial. Rather than a lack of sophisticated ICU technology, a lack of understanding of the CC principles continues to be a major obstacle to good-quality care. Participation in international research and quality improvement efforts can help enhance care delivery. Efforts to create a global registry of worldwide adult ICUs are in process [20], and similar attempts are urgently needed for critically ill children.

The study is a secondary data analysis of an investigation with different objectives, thereby introducing important limitations. Centers with capabilities and interest likely were self-selected and thus may not be representative of the average facilities of the region. However, the fact that 39 centers from resource-limited centers have comparable resources and interest in multicentric clinical trials is encouraging and may help in fostering a clinical trial group composed of the interested centers. The information also is self-reported on the basis of perceptions, rather than the clinical records, so an element of bias may be present.

## Conclusions

Absolute CC services differ widely around the planet. Although institutions in HICs clearly have more resources, LMICs have a greater disease burden. This study highlights the differences while showing the similarity and availability of basic infrastructure for services and research. Even after accounting for the limitations, this effort represents an important step to understand the resources available to sick children and for future research initiatives. A critical need exists for ongoing study of health services research in global pediatric CC and for optimization of research settings around the world to better care for the large proportion of the population who are currently affected but do not have access to services.

### Key messages

- First global survey on the resources and manpower in pediatric intensive care.
- Significant disease burden of common pediatric intensive care unit (PICU) conditions in low- and middle-income countries (LMICs).
- Availability of resources and manpower for clinical research exists in developing countries and is comparable to high-income countries at least in some centers.
- To generate data applicable to all the children worldwide, centers from LMICs need to be included in multicentric trials.

## Abbreviations

CC: critical care
CERTAINp: Checklist for Early Recognition and Treatment of Acute Illness in Pediatrics
HIC: high-income country
ICU: intensive care unit
LIC: low-income country
LMIC: low- and middle-income country
UMIC: upper-middle-income country
